# Supplementary material for: Auxin Transporter OsPIN1b, a Novel Regulator of Leaf Inclination in Rice (Oryza sativa L.)
Source: Plants (Basel). 2023 Jan 15;12(2):409. doi: 10.3390/plants12020409 (PMC9861231; doi:10.3390/plants12020409)
Supplement: Supplementary file 1 [file plants-12-00409-s001.zip › plants-2152127-supplementary.pdf]

Supplementary Material

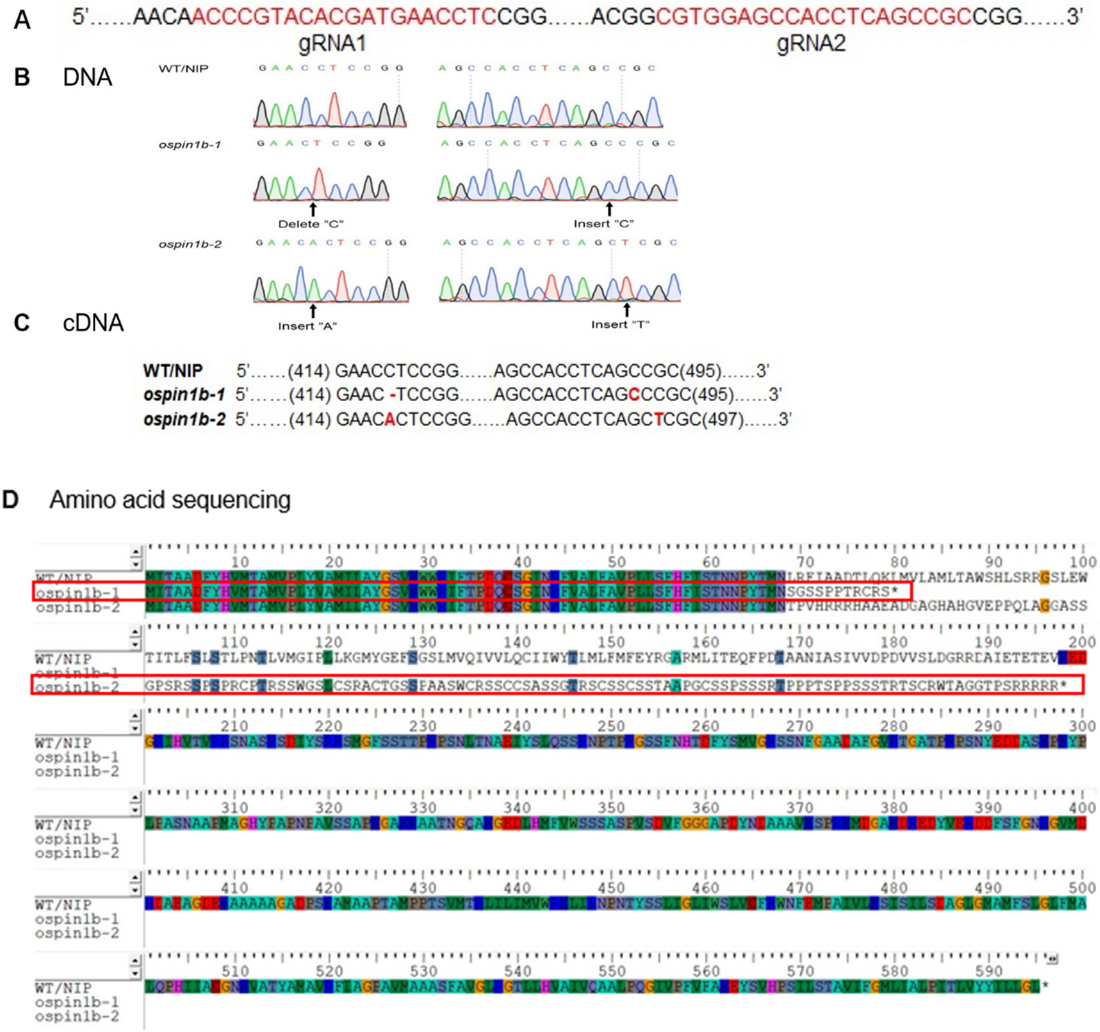

Figure S1. Construction of *ospin1b* mutants via CRISPR-Cas9 system. (**A**) Targeting sequence of gRNA1 or gRNA2. The red letters show targeting sequences. (**B**) DNA sequencing map of *OsPIN1b* gene in WT/NIP, *ospin1b*-1 and *ospin1b*-2. Black arrows point at the insertion and deletion sites. (**C**) cDNA sequences of *OsPIN1b* gene in WT/NIP, *ospin1b*-1 and *ospin1b*-2. The red letter indicates the inserted base, and the red dotted line indicates deleted base. (**D**) Amino acid sequences of *OsPIN1b* in WT/NIP, *ospin1b*-1 and *ospin1b*-2. The red frames are used for marking the altered amino acid sites after CRISPR-Cas9 gene editing.

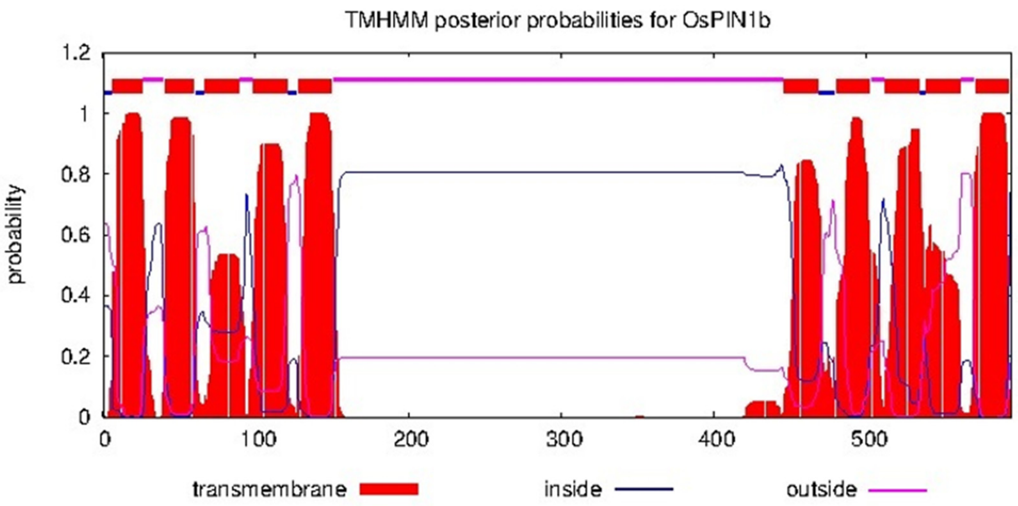

Figure S2. Structure analysis of OsPIN1b protein. Bioinformatic prediction of transmembrane region of OsPIN1b protein. The red rectangle, blue line and light purple line represent transmembrane, inside membrane, and outside membrane, respectively.

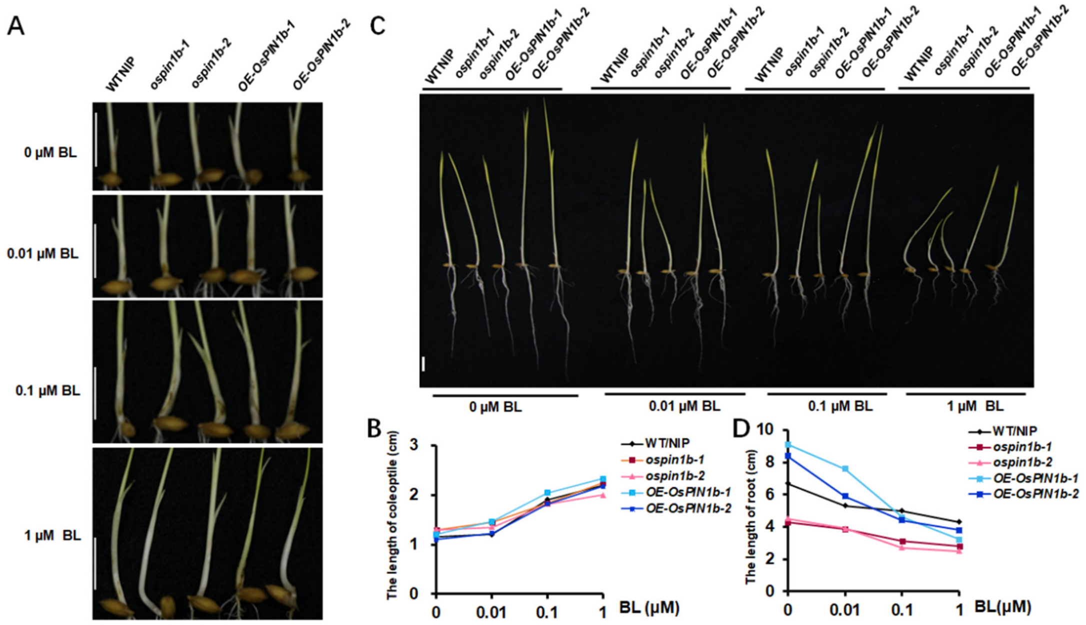

Figure S3. BR sensitivity tests. (A) Coleoptile response to 24-eBL treatment. The plant materials under each treatment were WT/NIP, *ospin1b-1*, *ospin1b-2*, *OE-OsPIN1b-1*, and *OE-OsPIN1b-2* from left to right; The concentrations of 24-eBL from top to bottom were 0, 0.01, 0.1 and 1μM, respectively. Scale bar = 1 cm. (B) statistical analysis of (A). The data are mean ± SD (n= 3). (C) Root response to 24-eBL treatment. Scale bar = 1 cm. (D) statistical analysis of (C). The data are mean ± SD (n= 3).

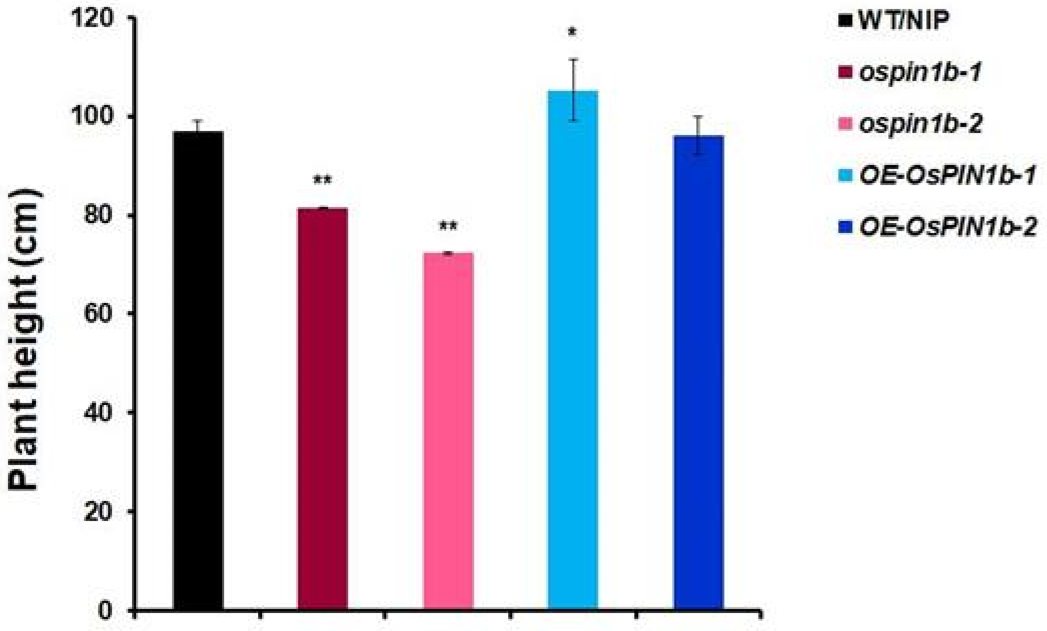

Figure S4. *OsPIN1b* positively regulates plant height. Statistics analysis of plant height of WT/NIP, *ospin1b* mutants and *OsPIN1b* over-expression lines for 4 months of seedling, respectively. The data are mean ± SD (n= 10) and * indicates the significant differences among WT/NIP, *ospin1b* mutants and *OE-OsPIN1b* over-expression lines (*$p < 0.05$, **$p < 0.01$; Student's *t*-test).

Table S1. Primers Used for Vector Construction.

Primer name	Primer sequence (from 5′ to 3′)
1b-gR1-FP	TA GGTCTCC ACGATGAACCTC gttttagagctagaa
1b-gR1-RP	AT GGTCTCA TCGTGTACGGGT tgcaccagccgggaa
1b-gR2-FP	TA GGTCTCC CACCTCAGCCGC gttttagagctagaa
1b-gR2-RP	AT GGTCTCA GGTGGCTCCACG tgcaccagccgggaa
L5AD5-FP	CGGGTCTCAGGCAGGATGGGCAGTCTGGGCAACAAAGCACCAGTG G
L3AD5-RP	TAGGTCTCCAAACGGATGAGCGACAGCAAACAAAAAAAAAAGCA CCGACTCG
S5AD5-FP	CG GGTCTCA GGCA GGATG GGCAGTCTG GGCA
S3AD5-RP	TA GGTCTCC AAAC GGATG AGCGACAGC AAAC
UGW-FP	GACCATGATTACGCCAAGCTTAAGGAATCTTTAAACATACG
UGW-RP	GGACCTGCAGGCATGCACGCGCTAAAAACGGACTAGC
promoter u3	TGGGTACGTTGGAAACCACG
pUBI10	GTTTGTTGGTCGCCGTTAGG
1b (1,2)-FP	GTCGTTCCACTTCATCTCCA
1b (1,2)-RP	CTCCCCGTACATGCCCTTG
1b-CDS-F	gagctcggtacccggggatccGGTACCATGATTACGGCGGCGGACTT
1b-CDS-R	gctcaccatgtcgacTCTAGACAGCCCAAGCAAGATGTAGT
1b-over-FP	TTCATGGCGCTGCAGCCGC
1b-over-RP	TGTACTCCTTGGCGAAGACGA
pro1b-GUS-FP	gtcgacCTCGCCATTTTTTAACTCGT
pro1b-GUS-RP	ggtaccCTTCGCCCCCCCTCTT

Table S2. Primers Used for qRT-PCR Analysis.

Primer name	Primer sequence (from 5′ to 3′)
Actin-FP	TGTATGCCAGTGGTCGTACCA
Actin-RP	CCAGCAAGGTCGAGACGAA
OsPIN1b-RT-FP	TGCACCCTAGCATTCTCAGCA
OsPIN1b-RT-RP	CCCTCCTCCCAAATTCTACTTC
BRI1-RT-FP	TACCAGAGCTTCAGATGCACCA
BRI1-RT-RP	AGTAGCTCAGGGTCGAAGACAT
D2-RT-FP	CCTTTTGGTGGTGGGCAGAG
D2-RT-RP	TGGGGAAGTTGACGATGTGGT
D11-RT-FP	TCATGGAAATGCCCAACAATT
D11-RT-RP	CCGAACGGCGTAAACTTCTT
ARF19-RT-FP	GTCCTACTGAATTTGTTATC
ARF19-RT-RP	CAGATAGAAACCCTATTTCG
IAA1-RT-FP	CTCGACTTCGAGGACACCGC
IAA1-RT-RP	GATGGTGAAGTGGGAGAAGAAC
